# COASTAL RETIREMENT: IT’S ALL FUN AND GAMES UNTIL THE HURRICANE HITS

**DOI:** 10.1093/geroni/igz038.1962

**Published:** 2019-11-08

**Authors:** Anne P Glass, Jenni Blair, Judith Nichols

**Affiliations:** University of North Carolina Wilmington, Wilmington, North Carolina, United States

## Abstract

Many people dream of retiring to the beach. Twenty-four individuals who retired from out-of-state to a beach area in southern North Carolina had previously been interviewed regarding their retirement process and decision to move to this destination. Hurricane Florence brought major flooding and devastation to the area in September 2018. Shortly after, 10 participants agreed to complete a second interview and data collection about their hurricane-related experiences. This sample consisted of 8 women and 2 men, average age of 74.4 (range=68-88), and all were white. Nine evacuated, including one who went to a shelter. This project provided a unique opportunity to compare answers about stress levels and how they felt about their choice to move to the area, before and after the storm. Six and five rated their stress high/very high just prior to, and during Florence, respectively, but stress levels returned to low/very low for 90%. Two stated the storm caused them to rethink their decision to move; one now says she feels ambiguous about her move and would probably not choose it again. Stress caused by uncertainty was a thread across all interviews. Anxiety and concern were experienced, but no one reported fear. Neighbors played an important role pre-, during, and post-storm. No participants had significant damages, although one had a break-in; all expressed gratitude. They reported some lessons learned to apply the next time. These findings will be of interest to planners and others. They also demonstrate the resilience of older adults in dealing with natural disasters.

